# Isolation and Characterization of Photosensitive Hemolytic Toxins from the Mixotrophic Dinoflagellate *Akashiwo sanguinea*

**DOI:** 10.3390/md23040153

**Published:** 2025-03-31

**Authors:** Jiling Pan, Ting Fang, Shuang Xie, Ning Xu, Ping Zhong

**Affiliations:** Institute of Hydrobiology/Key Laboratory of Eutrophication and Red Tide Prevention of Guangdong Higher Education Institutes, Jinan University, Guangzhou 510632, China; jnupjl@gmail.com (J.P.); tingfancy@163.com (T.F.); xxieshuang@163.com (S.X.)

**Keywords:** harmful algal bloom, hemolytic activity, photosensitive toxin, photosynthetic pigment, chromatography, mass spectrometry

## Abstract

The mixotrophic dinoflagellate *Akashiwo sanguinea* is known to have acute toxic effects on multiple marine organisms, while the composition and chemical properties of its toxins remain unclear. In this study, we established a method for separation and purification of *A. sanguinea* toxins using chromatographic techniques. The acetone extract of *A. sanguinea* exhibited higher hemolytic activity and shorter incubation time compared to methanol and ethyl acetate extracts. Five fractions were obtained by solid-phase extraction (SPE), of which SPE3 (acetone/water ratio 3:2) and SPE4 (acetone/water ratio 4:1) exhibited the highest hemolytic activities and allelopathic effects. Further purification on SPE3 and SPE4 using reverse-phase high-performance liquid chromatography (RP-HPLC) coupled with a diode array detector (DAD) resulted in 11 subfractions, among which Fr4-5 displayed the strongest hemolytic activity. Nearly all active subfractions exhibited higher hemolytic activities incubated under light than those in the dark (*p* < 0.05), suggesting that *A. sanguinea* can produce both photosensitive and non-photosensitive toxins, with the former being the primary contributors to its hemolytic activity. Molecular characterization by UV-Vis, FTIR, and HRMS/MS analysis revealed that the structural features of Fr4-5 were highly consistent with porphyrin analogs and could be derived from chlorophyll *c*-related precursors. These findings highlight that the photosensitive toxins in *A. sanguinea* may serve dual roles in stress adaptation and ecological competition, potentially contributing to the formation of the blooms.

## 1. Introduction

The mixotrophic dinoflagellate, *Akashiwo sanguinea* (also known as *Gymnodinium splendens* Lebour or *Gymnodinium sanguineum* K. Hirasaka) [1] has been described as a eurythermal (10–30 °C) and euryhaline (5–40 psu) species with a wide distribution range [2]. Since the first report in Japan [3], *A. sanguinea* has frequently triggered algal blooms worldwide, caused mortality of aquatic animals, and resulted in significant economic losses [4,5,6,7,8]. In China, *A. sanguinea* is one of the most frequently reported HAB (harmful algal bloom) species, with recurrent occurrences documented along coastal waters, including Hong Kong [9,10], Xiamen [11], and Yantai [12]. Recent studies have revealed that *A. sanguinea* can form cysts to survive unfavorable conditions [13], a survival strategy that may contribute to its global expansion and frequent bloom formation [14,15,16].

The specific mechanism by which *A. sanguinea* exerts harmful effects on marine organisms remains unclear. An earlier study noted that blooms of this species may kill fish and invertebrates mainly through oxygen depletion resulting from cell death and decay [17]; the production of low concentrations of reactive oxygen species (ROS) by *A. sanguinea* [18], along with the mucous secreted through its sheath pores [19], may also serve as contributing factors to the mortality of aquatic organisms. However, increasingly, studies have indicated that *A. sanguinea* possesses the ability to produce toxins and exert potential toxicity on aquatic organisms across trophic levels [20]. Laboratory studies have shown that *A. sanguinea* can release allelopathic compounds capable of inhibiting or lysing co-occurring phytoplankton during its growth phase [15,20]. Whole cells or cell lysates of *A. sanguinea* have exhibited acute toxic effect on aquatic animals, including zooplankton, shellfish, crustaceans, and finfish [15,21,22]. In addition, *A. sanguinea* cell extract also showed hemolytic activity against rabbit erythrocytes [20]. Due to its broad-spectrum toxicity to aquatic organisms, it is of great significance to clarify the composition and characteristics of *A. sanguinea* toxins.

In recent decades, algal toxins have been a hot issue in marine research. Different types of seafood toxin syndromes, including paralytic shellfish toxins (PST), diarrhetic shellfish toxins (DST), amnesic shellfish toxins (AST), neurotoxic shellfish toxins (NST), azaspiracid shellfish toxins (AZA), and ciguatera poisoning (CP), have received high attention and intensive research. These lipophilic and/or neurotoxic compounds demonstrate bioaccumulation potential in marine food webs, acting as efficient vectors for trophic transfer that pose significant threats to human health and marine ecosystems through both acute intoxication and chronic exposure pathways [23,24,25,26,27,28]. Hemolytic toxins produced by ichthyotoxic algal bloom species (e.g., *Karenia mikimotoi*, *Chattonella marina*) can cause large-scale mortality of fish, shellfish, and other marine animals during the blooms [29,30,31,32]. They can exert toxic effects on surrounding organisms, granting the toxin-producing organisms a competitive advantage, although the biochemical compositions of most hemolytic toxins are unknown.

The development of rapid and reliable toxin detection and analytical systems is pivotal for the isolation of novel toxins. This process typically employs hemolysis-guided fractionation coupled with sequential chromatography techniques (e.g., SPE, RP-HPLC) to purify bioactive compounds, followed by structural characterization using nuclear magnetic resonance (NMR) spectroscopy and high-resolution mass spectrometry (HRMS). To date, this integrated approach has enabled the isolation and structural elucidation of diverse hemolytic toxin classes with distinct architectures. Key examples encompass amphidinolides—macrolides initially isolated from *Amphidinium* spp. through hemolytic activity screening [33,34,35]—and karlotoxins from *Karlodinium veneficum*, which were first detected in 2002 but required a decade of LC-MS/MS method development to resolve their long-chain polyhydroxy-polyene architectures [36,37,38,39]. Similarly, the photosensitive porphyrin derivative H2-a from *Heterocapsa circularisquama* was definitively characterized through synergistic UV-Vis, HRMS, and NMR analysis [40]. Systematic purification and structural analysis of these metabolites not only elucidate their ecological roles in bloom succession but also unravel their molecular mechanisms of action, providing critical insights for mitigating harmful algal bloom impacts.

Although studies have demonstrated the significant toxicity of *A. sanguinea* toward multiple marine organisms, the composition and characteristics of its toxins remain unknown. In this study, hemolysis testing was used to guide fractionation, a series of chromatographic techniques were used for the fractionation, isolation, and purification of hemolytic toxins in *A. sanguinea*, and UV-Vis, FTIR, and HRMS/MS were used for molecular characterization. This study aims to establish a theoretical foundation for subsequent investigations into structure identification and toxin production mechanism.

## 2. Results

### 2.1. A. sanguinea Toxins

#### 2.1.1. Optimization of Extraction Solvent

**Figure 1:**
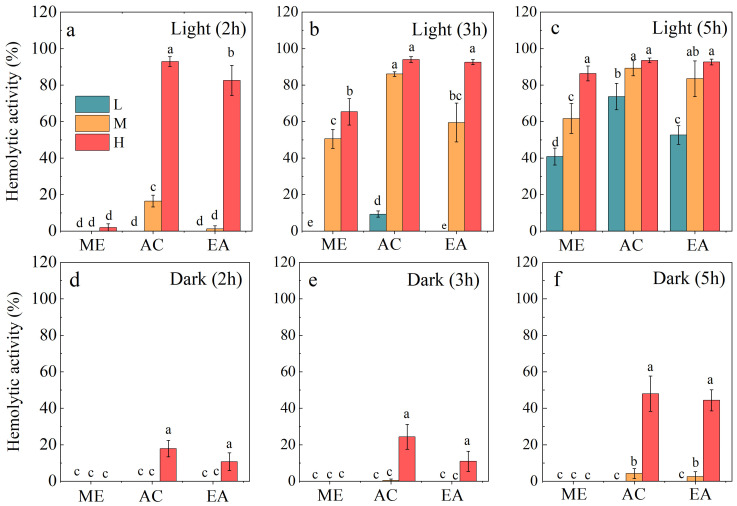
Hemolytic activities of *A. sanguinea* JX14 crude extracts using different organic solvents and incubated under light (**a**–**c**) or dark conditions (**d**–**f**). The incubation times were 2 h, 3 h, and 5 h. ME, AC, and EA represent methanol, acetone, and ethyl acetate, respectively. The corresponding cell densities of *A. sanguinea* were 100,000 cells/mL (L), 200,000 cells/mL (M), and 400,000 cells/mL (H), respectively. Results are expressed in triplicate ± standard deviation. Different lower-case letters indicate significant differences (*p* < 0.05).

Our results indicated that the cell-free culture filtrate of *A. sanguinea* exhibited negligible hemolytic activity, prompting us to focus solely on cell extracts for further analysis. Methanol, acetone, and ethyl acetate extracts of *A. sanguinea* cells demonstrated significant but markedly different hemolytic activities (Figure 1). Notably, hemolytic activity was significantly higher under light incubation compared to dark incubation, suggesting that *A. sanguinea* toxins contain photosensitive substances that play a primary role in driving hemolytic activity. The acetone extract showed the highest hemolytic activity, reaching 93% after 2 h of light incubation at maximum cell density, followed by the ethyl acetate extract at 83%, while the methanol extract exhibited negligible activity (Figure 1a). After five hours of light incubation, both acetone and ethyl acetate extracts achieved a comparable hemolytic activity of 94% (Figure 1c). In contrast, under dark conditions, the hemolytic activity of all extracts was significantly lower (*p* < 0.01). Additionally, hemolytic activity increased with prolonged incubation time and showed a positive correlation with cell density (r = 0.9364, *p* < 0.05). Among the solvents tested, acetone not only yielded the highest hemolytic activity but also required a shorter reaction time, making it the most effective extraction solvent. Therefore, acetone was selected for subsequent experiments to isolate and characterize the hemolytic toxins of *A. sanguinea*.

#### 2.1.2. Stability of *A. sanguinea* Toxins

(1) Thermal stability

**Figure 2:**
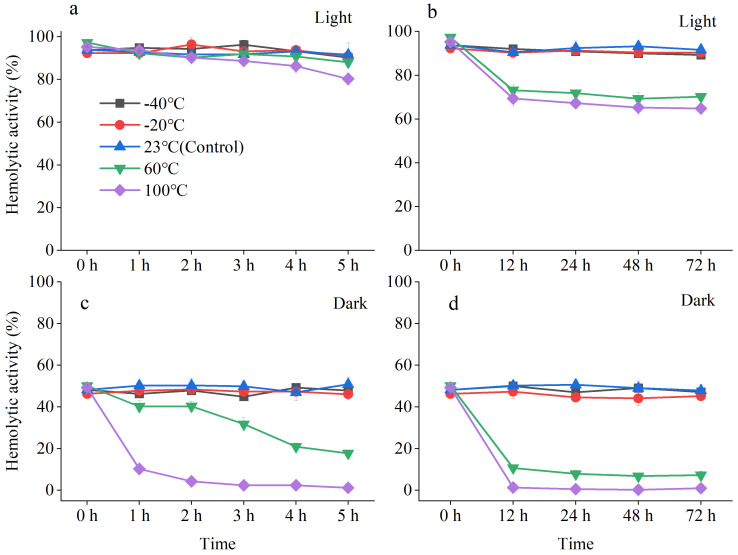
Hemolytic activities of acetone extract of *A. sanguinea* treated at different temperatures. (**a**): Short-term group under light incubation; (**b**): long time group under light incubation; (**c**): short-term group under dark incubation; (**d**): long-term group under dark incubation. The corresponding cell density of *A. sanguinea* was 4 × 10^5^ cells/mL.

Our results demonstrated that temperature strongly affected the hemolytic activity of *A. sanguinea* acetone extract, with notable differences in thermal tolerance between photosensitive and non-photosensitive components (Figure 2). Under dark-incubation conditions, the hemolytic activity reduced to nearly zero after one-hour treatment at 100 °C, whereas the hemolytic activity in both the room-temperature and low-temperature groups remained relatively constant over 72 h period (Figure 2c,d), indicating that the non-photosensitive toxins in *A. sanguinea* should be thermally unstable. In contrast, the hemolytic activity in the 100 °C treatment group under light incubation dropped by just 10% at 5 h (Figure 2a) and maintained at 70% at 72 h, despite a significant decline at 12 h (Figure 2b). There were no significant differences between the room-temperature and low-temperature treatment groups, whenever under dark incubation or light incubation (*p* < 0.05). This experiment demonstrated that, although photosensitive toxins may lose some of their activity at high temperatures, their overall stability is much higher than that of non-photosensitive toxins.

(2) Photostability

**Figure 3:**
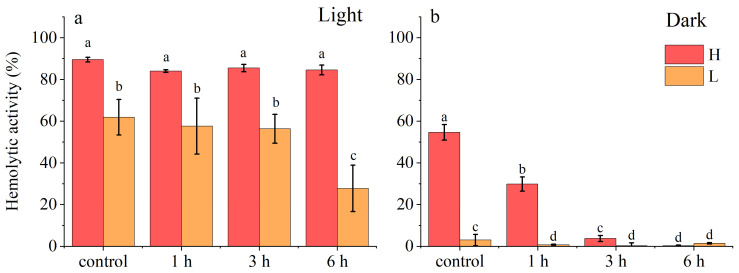
Hemolytic activities of acetone extract of *A. sanguinea* after light exposure at 100 µmol/(m^2^·s). (**a**): Light incubation; (**b**): dark incubation. The corresponding cell densities of *A. sanguinea* were 400,000 cells/mL (H) and 200,000 cells/mL (L). Results are expressed in triplicate ± standard deviation. Different lower-case letters indicate significant differences (*p* < 0.05).

Direct light exposure had a substantial impact on the hemolytic activity of *A. sanguinea* acetone extract, while a significant difference was found in the tolerance of photosensitive and non-photosensitive toxins (Figure 3). Under dark incubation conditions, the hemolytic activity of the high-density group decreased by over 50% after 1 h of light exposure and was nearly zero after 3 h. In contrast, under light incubation conditions, the hemolytic activity with the same cell density consistently reached approximately 90%, indicating that non-photosensitive toxins exhibited greater sensitivity to light exposure compared to photosensitive toxins. Under light incubation conditions, the hemolytic activity of the low-density group showed a significant decrease with 6 h of light exposure, indicating that light exposure in a short time (<3 h) has little effect on photosensitive hemolytic toxins. The lost hemolytic activity may mainly come from non-photosensitive hemolytic toxins; however, long-term light exposure (>6 h) will still significantly weaken the overall hemolytic activity of *A. sanguinea* toxins.

(3) Long-Term Chemical Stability

**Figure 4:**
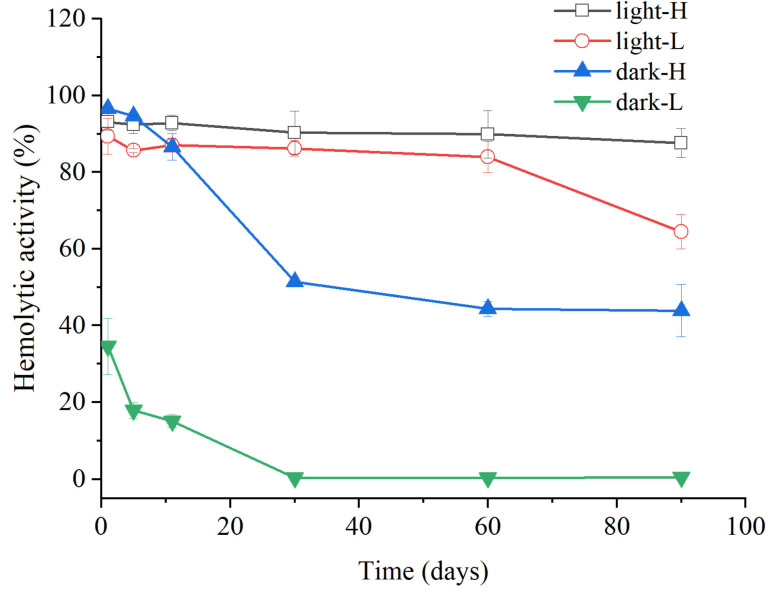
Stability of acetone extract of *A. sanguinea* (JX14) stored at −20 °C. The light and dark represent incubation conditions. The corresponding cell densities of *A. sanguinea* were 400,000 cells/mL (H) and 200,000 cells/mL (L). Results are expressed in triplicate ± standard deviation.

The trend of hemolytic activity of *A. sanguinea* extract stored at −20 °C over time is shown in Figure 4. Specifically, under dark incubation conditions, the hemolytic activity of the high-density group decreased by 50% after 30 days of storage, and the decrease was not significant between 30 and 90 days. This indicated that non-photosensitive hemolytic toxins contain compounds with differentiated chemical stabilities, and some components have good chemical stability. Under light incubation conditions, the hemolytic activity of the high-density group did not decrease significantly throughout the period, while the low-density group showed a significant decrease between 60 and 90 days, with a decrease of 20%. In general, the acetone extract of *A. sanguinea* was relatively stable under low temperature (−20 °C) storage conditions, and the chemical stability of photosensitive hemolytic toxins was better than that of non-photosensitive hemolytic toxins.

### 2.2. SPE Purification of A. sanguinea Acetone Extract

The acetone extract of *A. sanguinea* was eluted using five acetone/water (*v*/*v*) ratios (1/4, 2/3, 3/2, 4/1, 5/0) on the solid phase extraction (SPE) reverse C18 column, resulting in five elution fractions designated as SPE-1 to SPE-5. To investigate the active components, we assessed the hemolytic and allelopathic activities of the five SPE fractions.

#### 2.2.1. Hemolytic Activity of SPE Fractions

**Figure 5:**
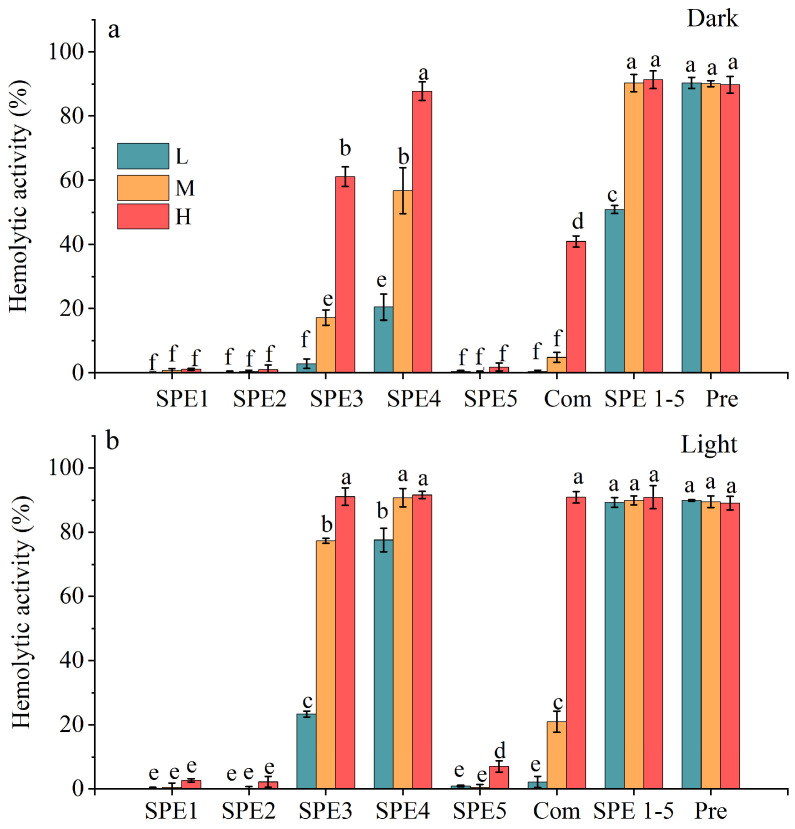
Hemolytic activity of SPE fractions of *A. sanguinea* JX14. The corresponding cell densities of *A. sanguinea* were 800,000 cells/mL (L), 1,200,000 cells/mL (M), and 1,600,000 cells/mL (H). (**a**): Dark incubation (5 h). (**b**): Light incubation (5 h). Combination (Com) represents a mixture of 200 µL of each SPE fraction; SPE1-5 represents a mixture of 1 mL of each SPE fraction, dried under a nitrogen stream and redissolved in 1 mL of ethanol; Pre represents acetone extract of *A. sanguinea* cells. Results are expressed in triplicate ± standard deviation. Different lower-case letters indicate significant differences (*p* < 0.05).

The hemolytic activity of different SPE components of *A. sanguinea* (JX14) was significantly different (Figure 5). Among them, SPE3 and SPE4 exhibited significantly higher hemolytic activity compared to the other fractions, regardless of incubation conditions, suggesting that the hemolytic compounds were primarily concentrated in these two fractions. Notably, under light incubation conditions, the hemolytic activity of SPE3 and SPE4 at each concentration was significantly higher than that under dark incubation conditions (*p* < 0.05), indicating the presence of both photosensitive and non-photosensitive toxins in these fractions. Furthermore, the hemolytic activity of the SPE fractions showed a clear density-dependent effect, with higher-density groups generally demonstrating greater hemolytic activity than lower-density groups. However, no synergistic effect was observed among the SPE fractions, as the hemolytic activity in the combination group was significantly lower than that of SPE3 and SPE4 individually (*p* < 0.05), except in the highest-density group under light incubation.

#### 2.2.2. Allelopathic Effect of SPE Fractions

**Figure 6:**
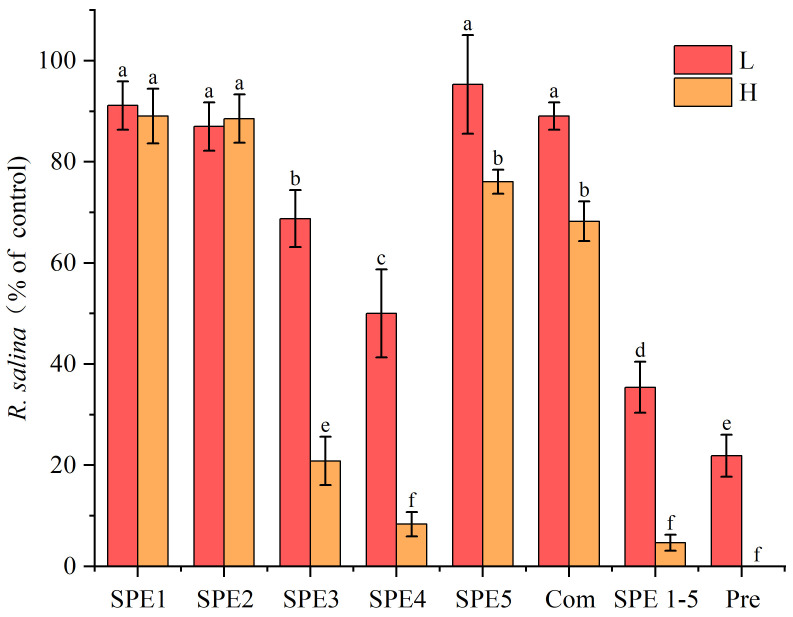
Inhibitory effect of SPE fractions of *A. sanguinea* on *R. salina* at 72 h. The corresponding cell densities of *A. sanguinea* were 2000 cells/mL (L) and 4000 cells/mL (H), respectively. Com, SPE1-5, and Pre are the same as in Figure 5. Results are expressed in triplicate ± standard deviation. Different lower-case letters indicate significant differences (*p* < 0.05).

*Rhodomonas salina* was used as target alga in this study due to its high sensitivity to allelopathic compounds [41]. All SPE fractions showed an allelopathic inhibitory effect on the growth of the cryptophyte *R*. *salina* after 72 h of treatment (Figure 6). Among these, SPE3 and SPE4 demonstrated significantly stronger inhibitory effects compared to the other fractions (*p* < 0.05), reducing *R. salina* densities to 21% and 8% of the control, respectively. The inhibitory effect displayed a clear density-dependent pattern. In general, high-density treatment groups exhibited significantly greater growth inhibition on *R. salina* than their low-density counterparts, except for SPE1 and SPE2. For instance, in the SPE3 group, *R. salina* density in the low-density treatment group was 68% of the control, whereas in the high-density treatment group, it was reduced to just 21%. These findings suggested that the compounds responsible for the allelopathic effect are primarily concentrated in SPE3 and SPE4. Furthermore, the trends among SPE fractions observed in allelopathic effect were completely consistent with those seen in hemolytic activity. This indicated that the hemolytic toxins of *A. sanguinea* may also have allelopathic activity, or alternatively, that SPE3 and SPE4 contain both hemolytic toxins and other allelopathic substances.

### 2.3. HPLC Fractionation of SPE3 and SPE4

**Figure 7:**
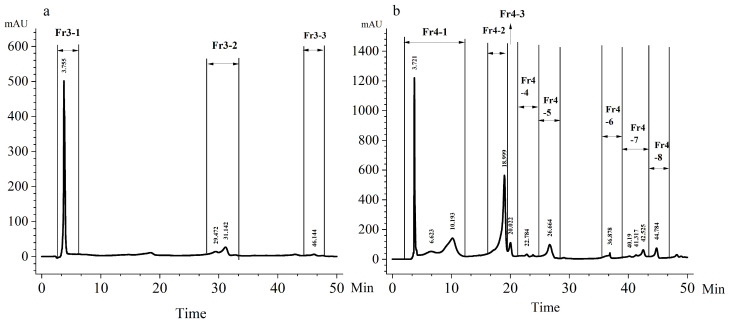
HPLC purification using SPE3 and SPE4. (**a**): HPLC purification using SPE3; (**b**): HPLC purification using SPE4. The scanning wavelength selected was 450 nm. The corresponding cell densities of SPE fractions of *A. sanguinea* were 4 × 10^7^ cells/mL.

The results of reversed-phase high-performance liquid chromatography (RP-HPLC) separation and purification using SPE3 and SPE4 fractions of *A. sanguinea* are shown in Figure 7. Three fractions (Fr3-1 to Fr3-3) were collected from SPE3 (Figure 7a), while eight fractions (Fr4-1 to Fr4-8) were obtained from SPE4 (Figure 7b) (indicated by arrows in the chromatogram).

**Figure 8:**
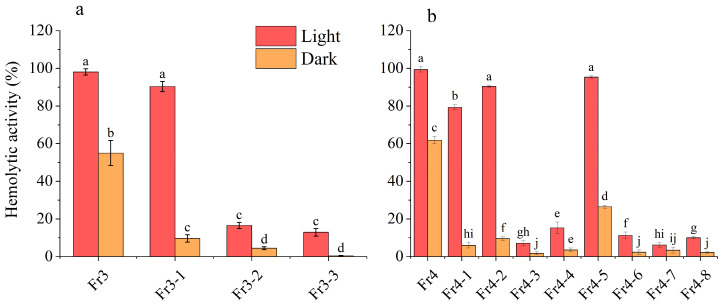
Hemolytic activities of HPLC fractions using SPE3 (Fr3) and SPE4 (Fr4). (**a**): Hemolytic activities of HPLC fractions from SPE3; (**b**): hemolytic activities of HPLC fractions from SPE4. The corresponding cell densities of HPLC fractions of *A. sanguinea* were 4 × 10^7^ cells/mL. Results are expressed in triplicate ± standard deviation. Different lower-case letters indicate significant differences (*p* < 0.05).

The purified product was evaluated for hemolytic activity, with the results presented in Figure 8. We found that *A. sanguinea* can produce a variety of different hemolytic toxins. The toxins in SPE3 were primarily concentrated in Fr3-1, whereas the toxins in SPE4 were predominantly found in Fr4-1, Fr4-2, and Fr4-5, with Fr4-5 exhibiting the highest hemolytic activity. Obviously, the SPE4 fraction contains more compounds with high hemolytic activity. Interestingly, all these hemolytic components seemed to be light-sensitive, as evidenced by the fact that their hemolytic activities were stronger in light than in darkness.

### 2.4. Characterization of HPLC Fraction Fr4-5

The HPLC-purified fraction Fr4-5, exhibiting the strongest hemolytic activity, was selected for subsequent analysis. Fr4-5 was subjected to additional reversed-phase high-performance liquid chromatography (RP-HPLC) purification under the conditions described in Materials and Methods Section 4.4 to harvest the samples required for high purity structural identification (final purity > 98%; HPLC-DAD spectral deconvolution, 200–800 nm).

#### 2.4.1. UV-Vis Analysis

**Figure 9:**
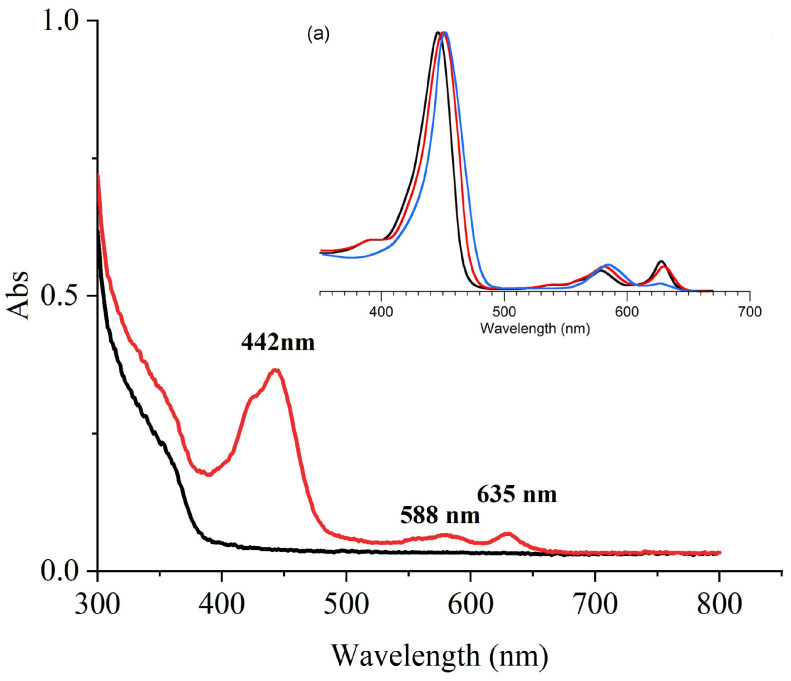
Comparison of UV-Vis absorption spectra of HPLC Fr4-5 and chlorophyll *c*. The corresponding cell densities of *A. sanguinea* were 4 × 10^7^ cells/mL. Absorption spectra of Fr4-5 in red, absorption spectra of anhydrous ethanol in black. (**a**) Absorption spectra (normalized) of chlorophyll *c*_1_ (black, in diethyl ether), chlorophyll *c*_2_ (red, in diethyl ether), and chlorophyll *c*_3_ (blue, in acetone) [46].

The UV-Vis absorption spectrum of fraction Fr4-5 is presented in Figure 9. Apart from the solvent-related absorption peak of anhydrous ethanol at 220 nm (excluded from the plotted spectral range), Fr4-5 displayed three characteristic absorption maxima at 442 nm (Soret band), 588 nm (Qx transition), and 635 nm (Qy transition). These spectral signatures are strongly indicative of a conjugated tetrapyrrole macrocyclic system, characteristic of porphyrinoids or chlorophyll-derived metabolites. The prominent Soret band at 442 nm corresponds to π→π* electronic transitions within the porphyrin chromophore framework, a hallmark of chlorophyll-related compounds [42]. The paired Q bands at 588 nm and 635 nm (Δλ = 47 nm) align with vibronic splitting patterns observed in metallated porphyrins [43]. Notably, the spectral profile closely matches reported data for chlorophyll *c*_1_ and chlorophyll *c*_2_ [44,45,46], suggesting structural homology between Fr4-5 and chlorophyll *c* derivatives.

#### 2.4.2. FTIR Analysis

**Figure 10:**
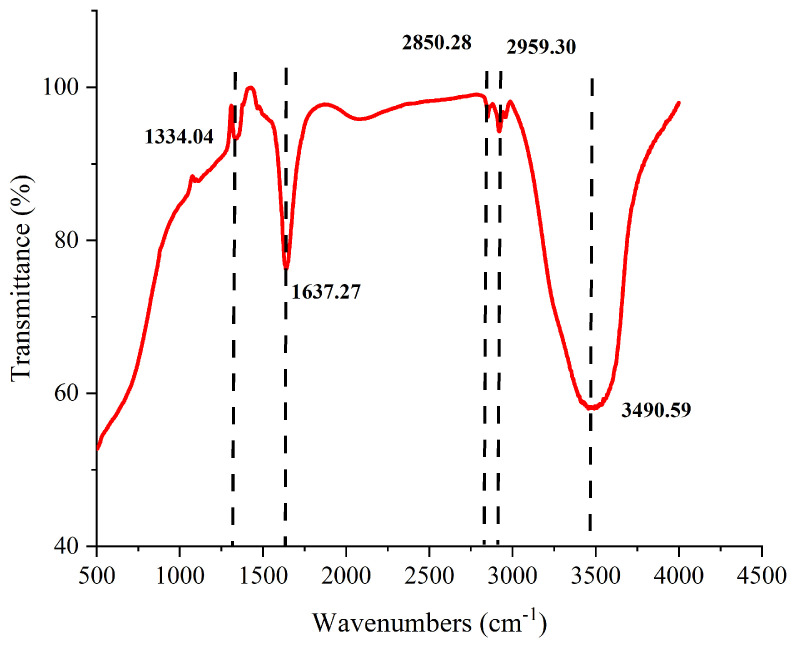
FTIR spectrum of HPLC fraction Fr4-5.

The Fourier transform infrared (FTIR) spectrum of fraction Fr4-5 displayed several prominent absorption peaks (Figure 10), revealing the presence of various typical functional groups within its molecular structure. The broad absorption peak at 3490 cm^−1^ is typically associated with the -OH stretching vibration, suggesting the presence of hydroxyl groups. The peaks at 2959 cm^−1^ and 2850 cm^−1^ are attributed to the C-H stretching vibrations, indicating the presence of alkyl chains or similar structural units. The peak at 1637 cm^−1^ is commonly associated with the bending vibration of C=C double bonds, suggesting the presence of aromatic rings or nitrogen-containing groups. The absorption at 1334 cm^−1^ may be attributed to C-N or C-H bending vibrations, which are typically observed in nitrogen-containing organic molecules. Overall, the absorption features in the spectrum indicated that the fraction Fr4-5 contained a range of typical organic functional groups, such as hydroxyl, alkyl, and aromatic or nitrogen-containing structures.

#### 2.4.3. HRMS/MS Analysis

High-resolution tandem mass spectrometry (HRMS/MS) in positive ion mode showed multiple peaks. The molecular ion peak *m*/*z* is observed to be 663.4545, speculated to be [M + H]^+^. Additionally, a minor peak at *m*/*z* 685.4352 was observed, corresponding to the [M + Na]⁺ adduct. These results suggest that the molecular weight of Fr4-5 may be 662. Additional fragment ions were detected at *m*/*z* 619.5273, 607.5661, and 579.5342, possibly corresponding to the loss of some functional groups in the compound (detailed fragment ion peaks and adduct ions are shown in Table S1).

## 3. Discussion

In this study, the hemolytic activity of *A. sanguinea* acetone extract and its purified components exhibited significantly greater potency under light exposure compared to dark conditions. This enhanced activity suggests that photochemical reactions mediated by light may either activate latent toxin structures or stabilize bioactive conformations. Our findings demonstrate that *A. sanguinea* synthesizes both photosensitive toxins (light-dependent toxic metabolites) and non-photosensitive toxins, with the former accounting for the predominant hemolytic effects. Specifically, these photosensitive compounds undergo wavelength-dependent transformations when irradiated with ultraviolet or visible light, generating hemolytic compounds through photoinduced radical formation and/or structural isomerization [47,48,49].

### 3.1. Stability of Hemolytic Toxins

Temperature plays a crucial role in determining the stability of bioactive substances. In this study, low-temperature treatments (−40 and −20 °C) had minimal impact on the hemolytic activity of *A. sanguinea*. In contrast, high-temperature treatments (60 and 100 °C) significantly reduced hemolytic activity (Figure 2). Interestingly, the effects of high temperatures differed between non-photosensitive and photosensitive toxins, with the non-photosensitive components highly sensitive to elevated temperatures, while the photosensitive components were remarkably thermally stable, tolerating temperatures up to 100 °C. Studies have shown that the thermal stability of algal toxins varies greatly. For example, cultures of *Cochlodinium polykrikoides* exhibited a loss of toxicity towards *Cyprinodon variegatus* following freezing or boiling treatment [50], whereas the fish-killing capability of *Alexandrium leei* cultures remained unaffected by these treatments [51].

The hemolytic activity of the crude extract from *A. sanguinea* was also significantly affected by light exposure. After a 3 h period of light treatment at 100 µmol/(m^2^·s), the hemolytic activity under dark incubation dropped to nearly zero, whereas the hemolytic activity under light incubation remained basically unchanged compared with the level before treatment (Figure 3). The observation suggested that the photosensitive components have higher photostability, while the non-photosensitive components may undergo degradation or structural alterations when exposed to light, resulting in reduced hemolytic activity. Similarly, studies have shown that the acute toxicity of cell-free filtrates of *P. parvum* to fish diminished markedly after a few hours of full sunlight exposure [52].

### 3.2. Molecular Characterization of Fraction Fr4-5

Based on the highest hemolytic activity observed during preliminary screening, the HPLC fraction Fr4-5 from the extract was prioritized for structural characterization. UV-Vis analysis revealed three distinct absorption maxima at 442, 588, and 635 nm (Figure 9), exhibiting close alignment with the characteristic spectral profile of chlorophyll c [44,45,53,54]. This spectral congruence strongly indicates the presence of chlorophyll *c*-type derivatives in Fr4-5. FTIR analysis further elucidated structural features (Figure 10): key vibrational bands at 3490 cm^−1^ (-OH stretching), 2959 cm^−1^ (aliphatic C-H stretching), 1637 cm^−1^ (aromatic/conjugated C=C stretching), and 1334 cm^−1^ (C-H/C-N bending), collectively indicating a porphyrin-type macrocyclic architecture bearing hydroxyl, alkyl, and aromatic functional groups. Moreover, HRMS/MS data corroborated these findings: the molecular ion peak at *m*/*z* 663.4546 ([M + H]⁺) corresponds to a molecular mass of 662 Da. Multidisciplinary validation provided compelling evidence that the photosensitive hemolytic agent in Fr4-5 represents a porphyrin analog. Although its characteristic absorption peak is highly consistent with chlorophyll *c*, the potential link with chlorophyll *c* precursors remains speculative rather than conclusive. This configuration, which has not been reported in dinoflagellate metabolomics studies, may reflect different biosynthetic pathways or oxidative remodeling of chlorophyll-like substrates and requires isotopic tracer studies to elucidate its biosynthetic origin.

### 3.3. Ecological Significance of Porphyrin-like Phycotoxins

The identification of a chlorophyll *c*-derived hemolytic agent aligns with historical reports of photosensitive porphyrin toxins in marine microalgae. In 2005, Miyazaki et al. isolated a porphyrin derivative with a molecular weight of 566 from the dinoflagellate *H. circularisquama* [40,55,56]. In the same year, Kuroda et al. isolated a highly photosensitive, porphyrin-like hemolytic toxin from the raphidophyte *C. marina*, with characteristic absorption peaks at 446, 583, and 635 nm [30], which closely match the characteristic absorption peaks of the HPLC fraction Fr4-5 of *A. sanguinea* in this study. Recent advances further implicate chlorophyll biosynthesis in toxin production: *C. marina* hemolytic activity correlates with chlorophyll *c*_2_ and chlorophyll *a* levels, implying toxin generation as a byproduct of chlorophyll *c*_2_-associated electron transport [57]. In addition, photosensitive toxins have also been observed in the dinoflagellate *Prorocentrum micans*, while research on these compounds remains sparse [30]. Our findings extend this paradigm to *A. sanguinea*, demonstrating that porphyrin analogs, presumably “retooled” chlorophyll intermediates, may represent a broad class of phycotoxins.

We propose an association mechanism between porphyrin accumulation and ecological adaptation in *A. sanguinea*. Under light limitation, phytoplankton typically upregulate chlorophyll synthesis to enhance photon capture [58], but this adaptation risks intracellular porphyrin intermediates accumulation. These intermediates may act as pro-oxidants, elevating ROS levels and inducing oxidative stress [59]. Conversion of excess porphyrins into hemolytic toxins could serve dual purposes: (1) mitigating ROS-mediated cellular damage and (2) conserving energy by repurposing metabolic intermediates, rather than catabolizing them, as a competition strategy, consistent with mixotrophic dinoflagellates’ reliance on toxin-enhanced heterotrophy under photosynthetic stress [60,61,62,63,64]. Importantly, coupling toxin synthesis to chlorophyll metabolism may confer a competitive advantage: by transforming a stress liability (porphyrin accumulation) into a chemical defense, *A. sanguinea* could suppress competitors while allocating resources for bloom formation. Further research on the stereo structural analysis by nuclear magnetic resonance spectroscopy (NMR) and targeted metabolomic and transcriptomic validation will provide new insights into toxins structure and production mechanism in *A. sanguinea*.

## 4. Materials and Methods

### 4.1. Algal Cultures Conditions

A clonal strain of *A. sanguinea* (JX14) was isolated from Daya Bay, Guangdong Province of China in 2011 (Genebank accession number KF793278). Strain *Rhodomonas salina* (CCMP1319) was provided by Professor Christopher J. Gobler from Stony Brook University, USA.

Algal strains were grown in silicate-free f/2 medium [65] with natural seawater. Cultures were maintained at constant temperature (20 °C) and irradiance (100 µmol/(m^2^·s)) in a 12 h light/12 h dark cycle. The seawater used was collected from Daya Bay and filtered through a 0.22 µm fiber filter before being sterilized in an autoclave.

### 4.2. Extraction of A. sanguinea Toxins

#### 4.2.1. Hemolytic Activity of *A. sanguinea* Extract

Cells extraction: Mid-exponential-phase (6 days after inoculation) *A. sanguinea* cultures (8000 cells/mL, 2000 mL) were harvested and centrifuged (1800× *g*, 10 min, 4 °C); algal cells were resuspended in acetone (100 mL). Then, ultrasonic cell disruption was performed in an ice bath (570 W, 10 min, 5 s/5 s), and the supernatant was collected after centrifugation (4000× *g*, 4 °C, 10 min). After microscopic examination, the cell-free supernatant was dried under a nitrogen stream and re-dissolved with ethanol (40 mL) to prepare the crude *A. sanguinea* cell extract with a corresponding cell density of 4 × 10^5^ cells/mL.

Filtrate extraction: Mid-exponential-phase (6 days after inoculation) *A. sanguinea* cultures (8000 cells/mL, 2000 mL) were harvested and centrifuged (1800× *g*, 10 min, 4 °C) to obtain the cell-free filtrate. Prior to use, the SPE column (Strata-X, 1 g/20 mL, Phenomenex, Torrance, CA, USA) was activated by the addition of 20 mL of methanol, followed by 20 mL of deionized (DI) water. The filtrate (20 mL) was then loaded onto a pre-activated SPE column, then eluted and collected with 20 mL acetone, blown dry under nitrogen flow, and then re-dissolved with ethanol (40 mL) to obtain the crude *A. sanguinea* filtrate extract with a corresponding cell density of 4 × 10^5^ cells/mL.

Both crude extracts were stored at −20 °C in an amber glass bottle in the dark for later analysis of hemolytic activity (see Section 4.6).

#### 4.2.2. Optimization of Extraction Solvent

The extraction effects of three organic solvents with different polarities were compared. Mid-exponential-phase *A. sanguinea* culture (6 days after inoculation, 8000 cells/mL, 2400 mL) was partitioned into three equal aliquots (800 mL) and centrifuged (1800× *g*, 4 °C, 10 min). The algal cells were collected and resuspended with methanol (50 mL), acetone (50 mL), and ethyl acetate (50 mL), respectively, then ultrasonically disrupted in an ice bath (570 W, 5 s/5 s, 10 min) and centrifuged (4000× *g*, 4 °C, 10 min). The supernatant was dried under a nitrogen stream and then redissolved in ethanol to obtain crude extracts of *A. sanguinea* with a cell density of 4 × 10^5^ cells/mL. The extracts were stored at −20 °C in the dark and subsequently tested for hemolytic activity (see Section 4.6).

#### 4.2.3. Stability of *A. sanguinea* Toxins

(1) Thermal stability

Acetone extracts of *A. sanguinea* (3 mL) were placed under five temperatures in the dark, including high temperature (60 and 100 °C), low temperature (−20 and 4 °C), and room temperature (23 °C). This experiment was comprised of two treatment cycles: a short-term group, where samples were collected every hour from 0 to 5 h, and a long-term group, where samples were collected every 12 h from 0 to 72 h.

(2) Photostability

Acetone extracts of *A. sanguinea* (1 mL) were exposed to light (100 µmol/(m^2^·s)) at 23 °C for 1 h, 3 h, or 6 h and followed hemolytic activity test (see Section 4.6). The acetone extract maintained at −20 °C in an amber glass bottle was used as a control.

(3) Long-term chemical stability

Acetone extract of *A. sanguinea* was prepared and stored at −20 °C in an amber glass bottle. An aliquot (1 mL) was removed on days 0, 1, 5, 11, 30, 60, and 90 for hemolytic activity testing (see Section 4.6).

### 4.3. SPE Purification of A. sanguine Acetone Extract

#### 4.3.1. SPE Fractionation from *A. sanguinea* Acetone Extract

C-18 SPE columns were employed to enhance the purification of the hemolytic toxins from acetone extract of *A. sanguinea*. Prior to use, the SPE column was activated by the addition of 20 mL of methanol, followed by 20 mL of deionized (DI) water. After the introduction of the sample (20 mL), the acetone extract of *A. sanguinea* was eluted using five acetone/water (*v*/*v*) ratios (1/4, 2/3, 3/2, 4/1, 5/0). Eluates were collected, evaporated by a rotary evaporator, and subsequently redissolved in ethanol (40 mL) to achieve a corresponding cell density of 4 × 10^5^ cells/mL. The SPE fractions were numbered SPE1 to SPE5 and preserved in amber glasses at −20 °C in the dark.

#### 4.3.2. Hemolytic and Allelopathic Activities of SPE Fractions

To isolate the active components for subsequent purification and to investigate possible combined effects among the SPE fractions, eight treatments were set up and tested for hemolytic and allelopathic activities (see Section 4.6). These treatments included groups SPE1 through SPE5, as well as groups Combination (a mixture of 200 µL of each SPE fraction), SPE1-5 (a mixture of 1 mL of each SPE fraction, dried under a nitrogen stream and redissolved in 1 mL of ethanol), and Pre (acetone extract of *A. sanguinea* cells).

### 4.4. HPLC Purification of SPE3 and SPE4

To further isolate and purify *A. sanguinea* toxins, SPE3 and SPE4 fractions were used for RP-HPLC analysis (Agilent 1100, Palo Alto, CA, USA) equipped with a diode array detector (DAD) and a reverse-phase column (Agilent 5 HC-C18, 250 mm × 4.6 mm, 5 µm). The detection wavelength was set at 450 nm. SPE3 and SPE4 fractions were concentrated to 4 × 10^7^ cells/mL and filtered through a 0.22-µm polycarbonate filter before use. The mobile phases consisted of deionized water (A) and methanol (B). A gradient elution program was applied at a flow rate of 0.8 mL/min, starting with 80% B, increasing to 90% B over 10 min, reaching 100% B at 50 min, and finally returning to the initial conditions. The injection volume was 10 µL, and the overall running time was 50 min. The column was equilibrated for 10 min between injections. Components from SPE3 and SPE4 were collected based on HPLC elution profiles, dried using rotary evaporation, and subsequently redissolved in anhydrous ethanol, resulting in a purified product with a final cell density of 4 × 10^7^ cells/mL. Hemolysis test was conducted using purified products (see Section 4.6).

### 4.5. Characterization of HPLC Fraction

#### 4.5.1. UV-Vis Analysis

The HPLC purified fraction Fr4-5 screened by hemolysis test was loaded in a 96-well plate (LABSELECT, Assay Microplate 12599, Anhui, China). UV-Vis spectrophotometer (BMG LABTECH, CLARIOStar, Ortenberg, Germany) was used to scan the sample in the range of 200–800 nm to determine the characteristic absorption peak of the hemolytic toxins. Anhydrous ethanol was used as the solvent control.

#### 4.5.2. FTIR Analysis

In this study, the KBr pellet method was used to measure the infrared absorption spectrum of the sample (Fr4-5). First, weigh 150 mg of dry potassium bromide (KBr) powder and compress it into transparent KBr tablets at room temperature through a tablet press. Use a capillary pipette to coat Fr4-5 on the KBr tablet and blow dry under nitrogen flow. During the coating process, ensure that the liquid is evenly distributed on the KBr tablets to obtain good spectral signals. Subsequently, testing was performed using infrared spectrometer (Thermo Fisher Scientific, Fisher Nicolet IS50, Waltham, MA, USA), measuring wave numbers ranging from 500 to 4000 cm^−1^. All tests were performed at room temperature, ensuring that the sample and instrument were stable before measurement. Infrared absorption spectrum data were collected and analyzed through instrument software to obtain the infrared characteristic peaks of the sample.

#### 4.5.3. HRMS/MS Analysis

High-resolution tandem mass spectrometry (HRMS/MS) was used to confirm the molecular weight range of *A. sanguinea* toxins. Mass spectrometric measurements were performed using a mass spectrometer (SCIEX, AB SCIEX 500R, Framingham, MA, USA) equipped with an ESI source, operated in positive-ionization mode, and a time-of-flight (TOF) analyzer. The injection volume was 200 μL. The capillary voltage and cone voltage were set to 3000 V and 30 V, respectively. The source temperature was maintained at 120 °C. The nebulizer gas flow was set to 350 L/h at 350 °C. The mass range of the instrument was set to *m/z* 50–1600, and the data were collected and analyzed using SCIEX OS software (version 3.31).

### 4.6. Hemolytic Activity and Allelopathic Activity Assays

#### 4.6.1. Hemolytic Activity

The hemolytic activity assay was performed according to methods described by Kuroda, Ou, and Eschbach [30,66,67]. The test was conducted in a centrifuge tube with a total volume of 1 mL, comprising 50 µL of sample (*A. sanguinea* acetone extract or fraction), 450 µL of rabbit blood buffer, and 500 µL of rabbit erythrocyte suspension at a concentration of 0.5% (The rabbit blood cells we used were purchased from Yuanye Biotechnology, with the product number S26628-100 mL, a concentration of 4%, and were produced in China). The mixed solutions were incubated at 23 °C under a light intensity of 50 µmol/(m^2^·s) or in darkness for 5 h. Then, it was centrifuged at 1200× *g* and 4 °C for 5 min, and 150 µL of the supernatant was transferred to a 96-well cell culture plate. The absorbance was measured at 414 nm using an ELISA reader.

An equal amount of ethanol or Triton X-100 was used to replace the sample as a solvent control and a positive control; an equal amount of rabbit blood buffer was used to replace rabbit erythrocyte to establish a background control.

The hemolysis percentage was calculated according to the following formula (1).

(1) $$H\%=\frac{E_{414}-A_{414}-N_{414}}{P_{414}-A_{414}}$$

*E*_414_ is sample absorbance, *A*_414_ is solvent control absorbance, *N*_414_ is background control absorbance, and *P*_414_ is positive control absorbance.

#### 4.6.2. Allelopathic Activity

A microalgal bioassay system was established in a 6-well culture plate with a total volume of 10 mL to quantify allelopathic activity of SPE fractions of *A. sanguinea* on the target alga, *R. salina*. SPE fractions were added into 6-well plates containing exponential-phase *R. salina* (cell density: 2000 cells/mL) enriched with silicate-free f/2 medium. The corresponding cell densities of SPE fractions were 4000 cells/mL (high) or 2000 cells/mL (low). In the solvent control, the SPE fractions were replaced with an equal volume of ethanol. *R. salina* was cultured in silicate-free f/2 medium as a blank control. After 72 h, samples were fixed in 2% Lugol’s iodine solution, and cell densities of *R. salina* were counted under a light microscope (Olympus Corporation, Olympus CX41, Tokyo, Japan).

### 4.7. Data Analysis

All experimental groups were conducted in triplicate, and the results were expressed as the mean ± standard deviation of three independent experiments. Data were analyzed using one-way analysis of variance (ANOVA), with statistical significance set at *p* < 0.05. Prior to ANOVA, tests for normality and homogeneity of variance were performed. Pearson’s correlation test was used to analyze the relationship between cell density and hemolytic activity. Statistical analyses were conducted using SPSS software (version 19) (IBM, New York, NY, USA), and data visualization was performed with Origin software (version 9) (OriginLab Corporation, Northampton, MA, USA).

## 5. Conclusions

Species of *A. sanguinea* can produce both photosensitive and non-photosensitive toxins, with the photosensitive components playing a predominant role in their hemolytic activity. The results of this study support that the main photosensitive toxin of *A. sanguinea* is a porphyrin analog, which may be related to the photosynthetic pigment (chlorophyll *c*) synthesis pathway. Its structural specificity may provide important lead compounds for the development of new photodynamic therapy drugs.

## Data Availability

Data are contained within this article.

## Supplementary Materials

The following supporting information can be downloaded at: https://www.mdpi.com/article/10.3390/md23040153/s1, Figure S1: HRMS spectrum of Fr4-5 showing [M + H]^+^ ions, acquired at 10 eV collision energy in positive mode; Figure S2: Flow chart of isolation, separation, and characterization of *Akashiwo sanguinea* toxins; Table S1: Fragment ion peaks from the MS/MS spectrum of [M + H]^+^, obtained at a collision energy of 35 eV in positive mode.
