# Supplementary material for: Isolation and Characterization of Photosensitive Hemolytic Toxins from the Mixotrophic Dinoflagellate Akashiwo sanguinea
Source: Mar Drugs. 2025 Mar 31;23(4):153. doi: 10.3390/md23040153 (PMC12028677; doi:10.3390/md23040153)
Supplement: Supplementary file 1 [file marinedrugs-23-00153-s001.zip › marinedrugs-3541648-supplementary.pdf]

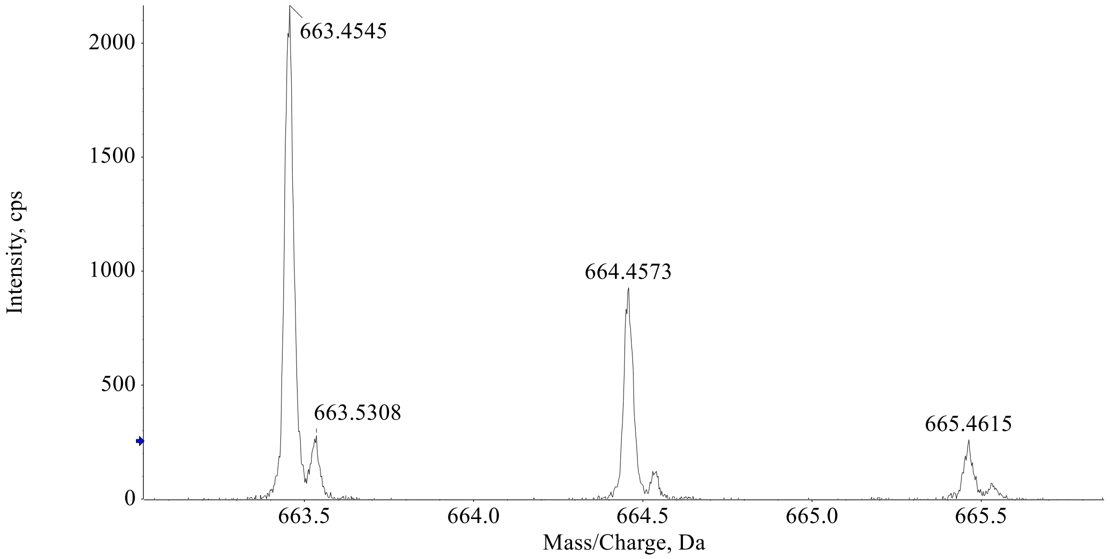

Figure S1 HRMS spectrum of Fr4-5 showing $[M+H]^+$ ions, acquired at 10 eV collision energy in positive mode.

Table S1 Fragment ion peaks from the MS/MS spectrum of $[M+H]^+$, obtained at a collision energy of 35 eV in positive mode.

Primary Ion	*m/z*	Fragment Ion	*m/z*
$[M+H]^+$	663.4545	607.5661	$[M+H -56]^+$
		619.5273	$[M+H -44]^+$
		645.4422	$[M+H -18]^+$
		579.5342	$[M+H -84]^+$

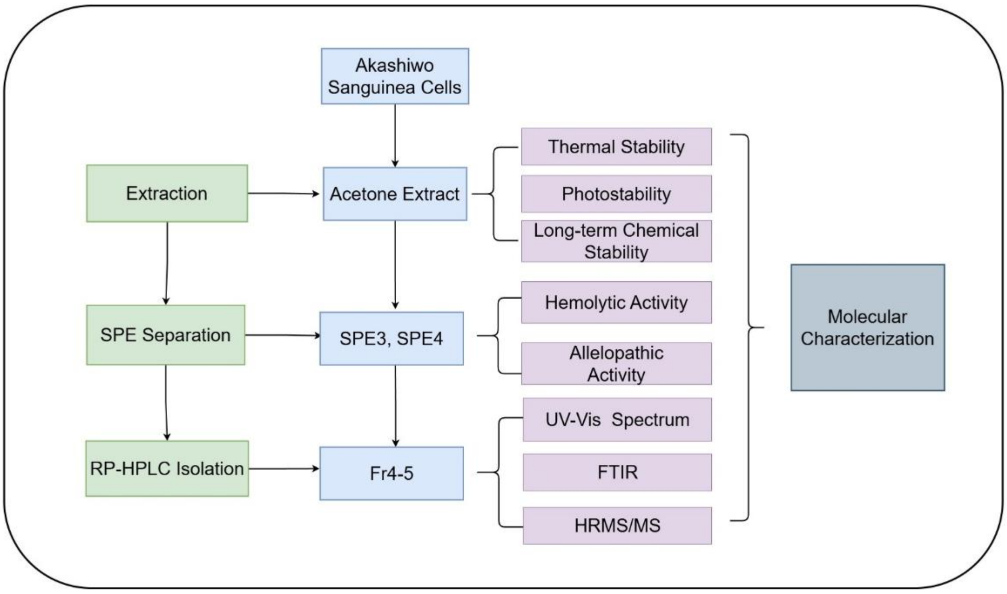

Figure S2 Flow chart of isolation, separation, and characterization of *Akashiwo sanguinea* toxins.
